# Regulation of Pituitary Cocaine- and Amphetamine-Regulated Transcript Expression and Secretion by Hypothalamic Gonadotropin-Releasing Hormone in Chickens

**DOI:** 10.3389/fphys.2019.00882

**Published:** 2019-07-25

**Authors:** Chunheng Mo, Can Lv, Long Huang, Zhengyang Li, Jiannan Zhang, Juan Li, Yajun Wang

**Affiliations:** ^1^Key Laboratory of Bio-resources and Eco-environment, Ministry of Education, College of Life Sciences, Sichuan University, Chengdu, China; ^2^Key Laboratory of Birth Defects and Related Diseases of Women and Children, Ministry of Education, West China Second University Hospital, Sichuan University, Chengdu, China

**Keywords:** pituitary, cocaine- and amphetamine-regulated transcript, hypothalamus, gonadotropin-releasing hormone, chicken

## Abstract

There is increasing evidence that cocaine- and amphetamine-regulated transcript (CART) peptide is abundantly expressed in the anterior pituitary of birds and mammals, suggesting that CART peptide may be a novel pituitary hormone and its expression and secretion is likely controlled by the hypothalamic factor(s). To substantiate this hypothesis, using chicken as an animal model, we examined the effects of gonadotropin-releasing hormone (GnRH) on pituitary CART secretion and expression and investigated whether GnRH could modulate plasma CART levels. The results showed that: (1) chicken GnRH (GnRH1 and GnRH2) could potently stimulate CART peptide secretion in intact pituitaries incubated *in vitro*, as detected by Western blot; (2) GnRH could also stimulate *CART* mRNA expression in cultured pituitary cells, as revealed by quantitative real-time polymerase chain reaction (qPCR) assay; (3) GnRH actions on pituitary CART expression and secretion are likely mediated by GnRH receptor coupled to the intracellular Ca^2+^, MEK/ERK, and cAMP/PKA signaling pathways; and (4) plasma CART levels are high in chickens at various developmental stages (1.2–3.5 ng/ml) and show an increasing trend towards sexual maturity, as detected by enzyme-linked immunosorbent assay (ELISA). Moreover, plasma CART levels could be significantly induced by intraperitoneal administration of GnRH in chicks. Taken together, our data provide the first collective evidence that CART peptide is a novel pituitary hormone and its expression and secretion are tightly controlled by hypothalamic GnRH, thus likely being an active player in the hypothalamic-pituitary-gonadal (HPG) axis.

## Introduction

It is well documented that cocaine- and amphetamine-regulated transcript (CART), an anorectic peptide of 41- or 48-amino acids with three intra-molecular disulfide bonds, plays an important role in the regulation of feeding and energy balance in mammals (Rogge et al., 2008). CART peptide encoded by *CART* gene was first discovered in abundance in the rat hypothalamus, in which it may act as a downstream effector of leptin with potent appetite-suppressing activity (Douglass et al., 1995; Gautvik et al., 1996; Kristensen et al., 1998; Elias et al., 2001). Besides the hypothalamus, CART is also expressed in other brain regions, the peripheral nervous system (PNS) and peripheral tissues of mammals including the anterior pituitary, ovary, and pancreas (Koylu et al., 1997; Jensen et al., 1999; Thim et al., 1999; Murphy et al., 2000; Kobayashi et al., 2004; Wierup et al., 2006; Ma et al., 2016), and is suggested to be involved in the regulation of many other physiological processes, such as drug reward and reinforcement, stress, pancreatic secretion, bone remodeling, and ovarian follicle development and steroidogenesis (Kobayashi et al., 2004; Elefteriou et al., 2005; Sen et al., 2007; Rogge et al., 2008; Abels et al., 2016).

Besides being a local regulatory factor found in the central nervous system (CNS) and peripheral tissues, CART peptide is present in the blood at physiological levels in mammals. In humans, the levels of plasma CART were reported to be higher in patients with neuroendocrine malignancy (Bech et al., 2008). In rats and rhesus macaques, the levels of circulating CART display diurnal variations, which is partially influenced by the circulating corticosteroids (Stanley et al., 2004; Vicentic et al., 2004). Moreover, blood CART levels are also related to the energy balance status, such as pregnancy/lactation in rats (Vicentic et al., 2004; Smith et al., 2006; Vicentic, 2006).

Despite the detection of physiological level of circulating CART in various species, its source has remained controversial. Several studies have suggested that the anterior pituitary may be a source of circulating CART peptide in mammals (Stanley et al., 2004). In rodents, CART mRNA and protein are found in the anterior pituitary (Thim et al., 1999; Murphy et al., 2000). More recent studies revealed that CART may be expressed in the gonadotrophs, lactotrophs, corticotrophs, thyrotrophs, and/or somatotrophs in rodents (Kuriyama et al., 2004; Stanley et al., 2004; Kappeler et al., 2006; Mortensen and Camper, 2016). In addition, pituitary CART secretion is reported to be under the control of hypothalamic factors such as corticotropin-releasing hormone (CRH) and circulating corticosterone (Stanley et al., 2004; Vicentic et al., 2004; Kappeler et al., 2006). Furthermore, intraperitoneal administration of CRH is demonstrated to increase plasma CART levels in rats (Stanley et al., 2004). Taken together, these findings tend to support the idea that CART is a pituitary hormone, which is released into the circulation under the influence of hypothalamic factor(s) and peripheral signal (Stanley et al., 2004; Kappeler et al., 2006). Regrettably, this notion has received little attention till now.

Recently, we have identified chicken *CART* gene (also named *CART1*) which is an ortholog of mammalian *CART* (Cai et al., 2015; Mo et al., 2015), and it encodes a mature CART peptide of 41 or 48 amino acids, which shows a striking homology (94–98% amino acid identity) with mammalian CART peptide (Cai et al., 2015). This suggests CART peptide may play important roles in chickens similar to their mammalian counterparts, such as inhibition of food intake (Tachibana et al., 2003; Honda et al., 2007). However, unlike mammalian CART abundantly expressed in the hypothalamus and pituitary (Rogge et al., 2008), chicken CART is predominantly and abundantly expressed in the anterior pituitary (Cai et al., 2015). This interesting finding led us to hypothesize that in chickens, CART peptide is a novel pituitary hormone, which expression/secretion is regulated by hypothalamic factors such as CRH demonstrated in our recent study (Mo et al., 2015). To further substantiate this hypothesis, using chicken as the animal model, our present study aims to: (1) investigate the effect of other hypothalamic factors, e.g., gonadotropin-releasing hormone (GnRH), on pituitary CART secretion and expression, and the involvement signaling pathways and (2) investigate whether GnRH affects plasma CART levels. Our findings represent the first to establish a clear concept that CART is a pituitary hormone, and its expression, secretion, and plasma levels are under the control of hypothalamic GnRH (GnRH1). Given that GnRH has a pivotal role in the reproduction of vertebrates including birds and mammals (Millar et al., 2004; Millar, 2005), our findings strongly suggest that CART peptide may also exert some influence on vertebrate reproduction.

## Materials and Methods

### Ethics Statement

Chickens were purchased from local commercial companies. All animal experiments were performed according to the guidelines provided by the Animal Ethics Committee of Sichuan University.

### Chemicals, Hormones, Antibodies, and Primers

Chicken GnRH1 and GnRH2 were synthesized using solid-phase Fmoc chemistry (GL Biochem, Shanghai, China). The pharmacological agents including 2-aminoethoxydiphenyl borate (2-APB), nifedipine, U73122, thapsigargin, KN62, calmidazolium, phorbol-12-myristate-13-acetate (PMA), calphostin C, PD98059, MDL12330A, and H89 were purchased from Calbiochem (Merck KGaA, Darmstadt, Germany). Rabbit anti-CART polyclonal antibody (CART-H47, sc366086) was purchased from Santa Cruz Biotechnology Inc. (Dallas, TX) and the specificity of anti-CART antibody was validated by Western blot/immunofluorescent detection of CART-specific bands/signals in chicken pituitary (but not in chicken liver, duodenum, and testes, which lack target gene expression) (Cai et al., 2015) or in HEK293 cells transfected with chicken CART expression plasmid (Cai, MSc thesis 2015). The monoclonal antibodies for β-actin, phospho-ERK1/2, ERK1/2, and phospho-CREB were purchased from Cell Signaling Technology Inc. (CST, Beverly, MA). Donkey anti-rabbit IgG (H+L) cross adsorbed secondary antibody (Dylight 488 conjugate) was purchased from ThermoFisher Scientific (Waltham, MA). The polyclonal antibodies against recombinant full-length chicken GH and PRL were prepared in our laboratory (Huang et al., 2014; Meng et al., 2014; Bu et al., 2016). All primers were synthesized by Beijing Genome Institute (BGI, China) and listed in Table 1.

**Table 1:** Primers used in this study.^a^

Gene	Sense/antisense	Primer sequence (5′- to -3′)	Size (bp)
**Primers for quantitative real-time RT-PCR assays**			
CART	Sense Antisense	CGTCCCGAGAGAAGGAGCTGATC ACTGCTCTCCGGCGTCGCACAT	123
LHβ	Sense Antisense	TGCGGCCCCATAGAGCCATG GTGGTGGTCACAGCCATACA	229
GnRHR1	Sense Antisense	GCATCACCCCAGCTATTTCTC GTGCCTTGGAGATGTGGTCAT	253
GnRHR2	Sense Antisense	TCGCTGTGCCGCAGCTGTTC CTGGAGGAGAAGAGGCTGGAGC	229
GH	Sense Antisense	CAAGCAACACCTGAGCAACT CAGCGTGACCACAGCGATGA	83
PRL	Sense Antisense	GCGGGTTCATTCTGGTGATGCT TGGATTAGGCGGCACTTCAAA	180
β-actin	Sense Antisense	CCCAGACATCAGGGTGTGATG GTTGGTGACAATACCGTGTTCAAT	123
**Primers used for RT-PCR assays**			
GnRHR1	Sense Antisense	GCATCACCCCAGCTATTTCTC GTGCCTTGGAGATGTGGTCAT	253
GnRHR2	Sense Antisense	TCGCTGTGCCGCAGCTGTTC CTGGAGGAGAAGAGGCTGGAGC	229
β-actin	Sense Antisense	TGTGCTACGTCGCACTGGAT GCTGATCCACATCTGCTGGA	401
**Primers for constructing expression plasmids**^b^			
GnRHR1	Sense Antisense	CCCAAGCTTCATGTGCGTACCAGCTGCT CCGGAATTCCTTCAGCACACCGTGTTAAC	1,149
GnRHR2	Sense Antisense	CCCAAGCTTACATGGCCCGGCTCGGCG CCGGAATTCGCTCACAGCGCACTGCTCTG	1,282

a *All primers were synthesized by BGI (China)*.

b *Restriction sites added in 5΄-end of the primers are underlined*.

### Quantitative Real-Time Polymerase Chain Reaction Assay

Chicken anterior pituitaries were collected either for cell culture, or for total RNA extraction. Total RNA was extracted from chicken tissues or cultured pituitary cells using RNAzol (Molecular Research Center, Cincinnati, OH) and reversely transcribed using M-MLV reverse transcriptase (TaKaRa). Reverse transcription (RT) samples were then used for quantitative real-time PCR (qPCR) assays to investigate the mRNA expression of target genes, as described in our recent studies (Cai et al., 2015; Mo et al., 2015).

### Detection of Cocaine- and Amphetamine-Regulated Transcript (CART) Expression in Chicken Pituitaries or Dispersed Pituitary Cells

Anterior pituitaries collected from adult male chickens were fixed in 4% paraformaldehyde and embedded in paraffin wax. Immunohistochemical staining was performed in the pituitary sections, as described in our recent studies (Huang et al., 2014; Bu et al., 2016). Anti-CART (1:300) was used to probe the spatial distribution of CART in anterior pituitaries, as previously described (Mo et al., 2015). Sections incubated with rabbit pre-immune serum, instead of anti-CART, were used as negative controls. To probe the spatial distribution of pituitary *CART* mRNA, the anterior pituitaries collected from adult male chickens were washed with PBS. The cephalic lobe and caudal lobe were separated carefully by scalpels, and their respective tissue lysates and total RNA were prepared for qPCR assay of *CART* (*LHβ*, *GnRHR2*, *GH*, or *PRL*) expression respectively, as described in our recent studies (Huang et al., 2014; Mo et al., 2015).

As described in our recent studies (Bu et al., 2016; Mo et al., 2017), the dispersed pituitary cells from adult male chickens were cultured in Medium 199 supplemented with 15% fetal bovine serum (Invitrogen) in a Corning Cell-BIND 48-well plates (Corning, Tewksbury, MA) at 37°C with 5% CO_2_ at a density of 5 × 10^5^ cells/well. After 4 h of culture, the pituitary cells were fixed in 4% paraformaldehyde and washed with PBS. Then, the cells were treated by 0.1% Triton-X-100 for 10 min, washed with PBS, and incubated with the blocking buffer (1% BSA in PBS) for 30 min at room temperature. After blocking, anti-CART (1:300), anti-GH (1:500), or anti-PRL (1:300) diluted in blocking buffer respectively, was added to each well and incubated at 4°C overnight. The cells were washed and incubated with the secondary antibody (1:300) for 1 h at room temperature. Finally, the pituitary cells were counterstained with 1 μg/ml DAPI and observed under a fluorescence microscope (Nikon ECLIPSE Ti).

### RNA-Seq Detection of CART, GnRHR1, and GnRHR2 mRNA Transcripts in Chick Hypothalamus and Anterior Pituitary

The mRNA levels of *CART*, *GnRHR1*, and *GnRHR2* were examined by analyzing our RNA-Seq data of the hypothalamus and anterior pituitary from 1-week-old male chicks (Mo et al., unpublished data). The quantification of reads was performed with Salmon v0.8.2 (Patro et al., 2017) against the Ensembl database^1^. The transcripts per million (TPM) values were used to estimate the abundance of *CART*, *GnRHR1*, and *GnRHR2* mRNA transcripts.

### Evaluation of GnRH1/GnRH2 Action on CART Secretion From Intact Pituitaries Incubated *in vitro*

As described in our recent study (Huang et al., 2014), the intact anterior pituitaries collected from 1-week-old male chicks were washed with PBS and placed on a 48-well plate (NUNC) supplemented with 400 μl serum-free Medium 199 (Invitrogen). After 1-h incubation at 37°C, the medium was replaced by 300 μl serum-free Medium 199 containing different concentrations of chicken GnRH1 or GnRH2 (0.1–100 nM) and incubated for 4 h. Then, CART peptide secreted into the incubation medium by the pituitaries was examined by Western blot using anti-CART antibody (1:300). Parallel blotting of β-actin and CART in pituitary tissue lysates was also conducted.

### Detection of CART Peptide Levels in Chicken Plasma by Enzyme-Linked Immunosorbent Assay

To evaluate plasma CART peptide levels in chickens, blood samples were obtained from the wing vein of male chickens (2-week-old, 5-month-old, and 15-month-old) and collected in the vacutainer blood collection tubes with K_2_EDTA. All samples were taken from each animal between 14:00 and 15:00. The blood samples were centrifuged at 1,600 × *g* for 15 min at 4°C, and plasma was collected and stored at −80°C until use. The levels of CART peptide in chicken plasma was determined by a commercially available competitive enzyme-linked immunosorbent assay (ELISA) kit (EK-003-61, Phoenix Pharmaceuticals, Inc.), which is designed to specifically detect the CART peptide based on the principle of competitive enzyme immunoassays.

To examine whether GnRH can elevate the plasma CART levels *in vivo*, male chicks (17-day-old) were intraperitoneally injected with 1 ml of saline for 2 days to acclimatize them to the injection procedure. On the following day, chicks (*n* = 7/group) were intraperitoneally injected with either saline or 5 μg/chick of GnRH1, and 30 min after the administration, blood was obtained from the wing vein of these chicks and collected in the vacutainer blood collection tubes with K_2_EDTA as soon as possible. The levels of CART peptide in chicken plasma was then determined by competitive ELISA assay.

### Investigation on the Effect of Chicken GnRH1/GnRH2 on CART Expression in Cultured Chick Pituitary Cells

As described in our recent studies (Huang et al., 2014; Mo et al., 2015), anterior pituitaries collected from 1-week-old chicks were sliced and digested by 0.25% trypsin at 37°C for 20 min. The dispersed pituitary cells were cultured at a density of 5 × 10^5^ cells/well in Medium 199 supplemented with 15% fetal bovine serum in a Corning Cell BIND 48-well plates (Corning, Tewksbury, MA) at 37°C with 5% CO_2_. After 24-h culture, the medium was replaced with serum-free M199 medium and the cells were treated with chicken GnRH1/GnRH2 for the duration (12–48 h) or with increasing doses (0.1–100 nM) of each peptide. The total RNA was then extracted from cultured pituitary cells and the expression of *CART* and *β-actin* assayed by qPCR.

### Functional Characterization of Chicken GnRHR1 and GnRHR2 in Cultured Chinese Hamster Ovary Cells

Based on the reported cDNA sequences of chicken *GnRHR1* and *GnRHR2* (accession nos.: NM_204653; NM_001012609) (Sun et al., 2001; Shimizu and Bedecarrats, 2006), gene-specific primers were used to amplify the open reading frame from adult chicken anterior pituitaries using high-fidelity Taq DNA polymerase (TOYOBO, Japan). The amplified PCR products were cloned into the pcDNA3.1 (+) expression vector (Invitrogen) and sequenced. According to our previously established methods, the signaling property of each receptor was examined in Chinese hamster ovary (CHO) cells using pGL3-NFAT-RE-luciferase (Wang et al., 2012), pGL4-SRE-luciferase (Mo et al., 2015), and pGL3-CRE-luciferase reporter systems (Wang et al., 2007).

### Data Analysis

The relative mRNA levels of *CART* (or other genes) in pituitary cells (or pituitary) were first calculated as the ratios to that of *β-actin* and then expressed as the percentage compared to their respective controls. The data were analyzed by the Student’s *t*-test (between two groups) or by one-way ANOVA followed by the Dunnett test in GraphPad Prism 5 (GraphPad Software, San Diego, CA). To validate our results, all experiments were repeated at least three times.

## Results

### Detection of Cocaine- and Amphetamine-Regulated Transcript (CART) Peptide in Chicken Anterior Pituitary and Plasma

**Figure 1:**
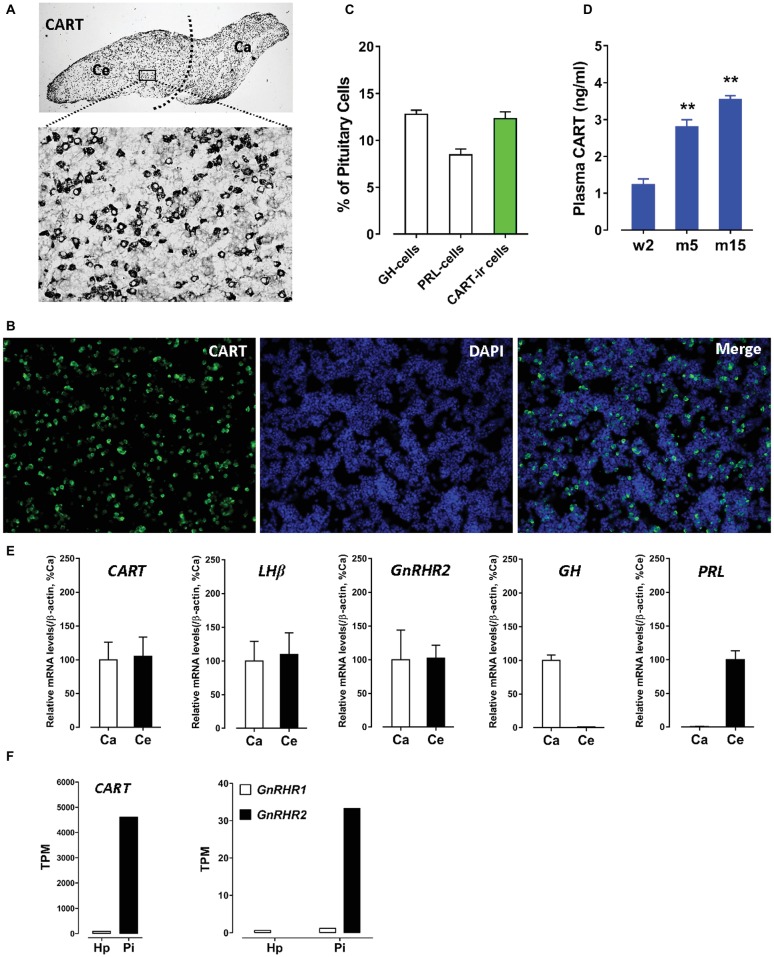
**(A)** Immunohistochemical staining of cocaine- and amphetamine-regulated transcript (CART) in the anterior pituitary of adult male chickens. Histological examination was performed under bright-field illumination with a magnification at 40× (upper panel) and 400× (lower panel). CART-ir cells were found to be densely distributed in the cephalic (Ce) and caudal (Ca) lobes of anterior pituitaries. **(B)** Immunofluorescence staining of CART signal (green) in short-term cultured dispersed pituitary cells of adult male chickens. **(C)** Graph shows the relative proportion (positive cells/DAPI-stained nuclei %) of GH-cells (~12.8%), PRL-cells (~8.5%), and CART-ir cells (~12.3%) in short-term cultured dispersed pituitary cells. Each data point in graphs represents mean ± SEM of four replicates (*N* = 4). FIGURE 1**(D)** Enzyme-linked immunosorbent assay (ELISA) assay of plasma CART peptide levels in male chickens at the stages of 2-week-old (w2), 5-month-old (m5), and 15-month-old (m15). ^**^p < 0.01 vs. w2. Each data point in graphs represents mean ± SEM of four individuals (*N* = 4). **(E)** Quantitative real-time PCR (qPCR) assay of *CART*, *LHβ*, *GnRHR2*, *GH*, and *PRL* mRNA levels in the Ce and Ca lobes of adult male chicken anterior pituitaries. Each data point represents mean ± SEM of 10 individuals (*N* = 10). **(F)** RNA-Seq analyses of *CART*, *GnRHR1*, and *GnRHR2* mRNA levels in the hypothalamus (Hp) and anterior pituitary (Pi) of 1-week-old male chicks.

Using immunohistochemical staining (IHC), we first examined the spatial distribution of CART in the anterior pituitary of adult male chicken. As shown in Figure 1A, CART-ir cells were densely distributed in cephalic (Ce) and caudal (Ca) lobes, as previously reported (Mo et al., 2015). Using immunofluorescence (IF) assay (Mo et al., 2017), we further revealed that CART-ir cells made up ~12.3% of the short-term cultured dispersed pituitary cells (Figures 1B,C). As a control, somatotrophs (GH cells) and lactotrophs (PRL cells) made up ~12.8 or ~8.5% of the cells under the same condition (Figure 1C).

The extremely high expression level of CART and the high proportion of CART-ir cells within the anterior pituitary led us to propose that like GH and PRL, CART can be released into the circulation. As expected, a high concentration (1.2–3.5 ng/ml) of CART was detected in the plasma of male chickens at different developmental stages by ELISA (Figure 1D). As shown in Figure 1D, a significant increase in plasma CART levels was noted from the pre-pubertal stage (2-week-old, 1.2 ng/ml) to sexually mature stages (5-month-old, 2.8 ng/ml; 15-month-old, 3.5 ng/ml). The remarkably high levels of plasma CART, together with the predominant expression of CART in anterior pituitaries, strongly support our hypothesis that CART is a pituitary hormone which is released into the circulation under the control of hypothalamic factors in chickens (Cai et al., 2015; Mo et al., 2015).

### Gonadotropin-Releasing Hormone (GnRH) Stimulates Pituitary CART Secretion and Elevates Plasma CART Levels

**Figure 2:**
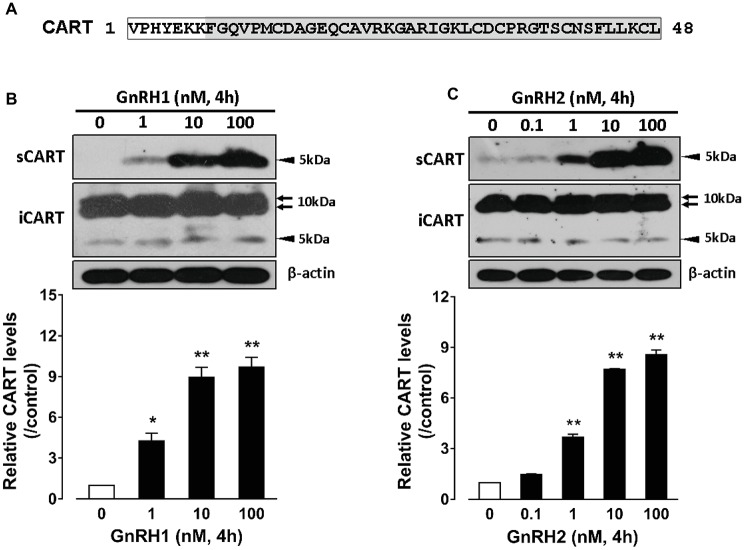
**(A)** Sequence of mature chicken cocaine- and amphetamine-regulated transcript (CART) peptide (~5 kDa) of 48 amino acids or 41 amino acids (shaded) cleaved from a large CART precursor of ~10 kDa (accession no.: KC249966) (Cai et al., 2015). **(B,C)** Western blot analyses showed that **(B)** chicken GnRH1 (1–100 nM, 4 h) and **(B)** GnRH2 (0.1–100 nM, 4 h) treatment could enhance the secretion of mature CART peptide into the incubation medium of chick anterior pituitaries incubated *in vitro*. The intracellular CART (iCART) and β-actin levels in the pituitary tissue lysate were also examined in parallel. The relative CART levels (sCART band, 5 kDa) in the incubation medium were quantified by densitometry, normalized by that of β-actin band in pituitary tissue lysate, and then expressed as fold increase of respective controls without GnRH1/GnRH2 treatment. Each data point represents mean ± SEM of three replicates (*N* = 3). ^*^p < 0.05; ^**^p < 0.01 vs. respective control. Note: the three major CART bands including the two large CART bands (~10 kDa) and a small band (~5 kDa) were identified in chick pituitary tissue lysate (iCART), whereas only the small band (~5 kDa) representing the mature CART was identified in the incubation medium (sCART) of pituitaries. One representative set of Western blot is shown at the top of each graph.

**Figure 3:**
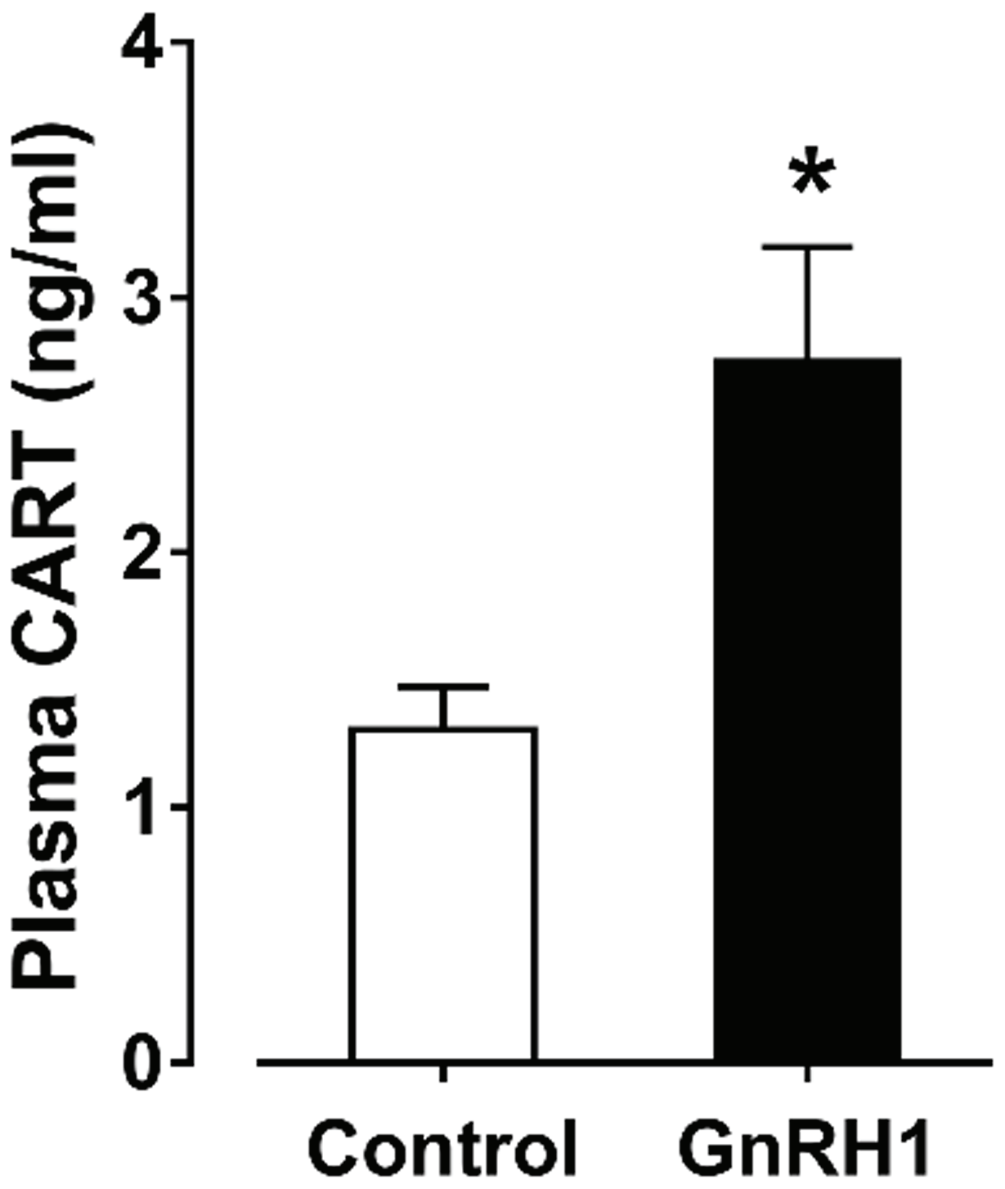
Enzyme-linked immunosorbent assay (ELISA) analyses showed that intraperitoneal administration of GnRH1 (5 μg/chick, 30 min) significantly elevated plasma cocaine- and amphetamine-regulated transcript (CART) peptide levels *in vivo*. Each data point represents mean ± SEM of seven 19-day-old male individuals (*N* = 7). ^*^p < 0.05 vs. saline (control).

Since the spatial distribution of *CART* detected by qPCR within anterior pituitaries is much similar to that of *LHβ* (Proudman et al., 1999; Scanes, 2015) and different from that of *GH*/*PRL* (Figure 1E), it led us to hypothesize that like pituitary LH (Sharp et al., 1986, 1987), CART secretion is likely controlled by the hypothalamic GnRH in chickens. To test this hypothesis, the effects of two chicken GnRHs (GnRH1 and GnRH2) on CART peptide release were examined both *in vitro* and *in vivo*. As shown in Figure 2, GnRH1 and GnRH2 (0–100 nM, 4 h) potently stimulated the secretion of mature CART peptide with a molecular weight of ~5 kDa (41/48 amino acids) in intact pituitaries incubated *in vitro*, with the minimal effective concentration observed at 1 nM, as revealed by Western blot. In agreement with this finding, intraperitoneal injection of GnRH1 (5 μg/chick, 30 min) caused a two-fold or higher increase (1.3 vs. 2.7 ng/ml, *p* < 0.05) in plasma CART levels *in vivo*, as monitored by ELISA (Figure 3). These findings clearly indicate that GnRH can potently stimulate pituitary CART secretion and elevate circulating CART levels effectively.

To determine whether the two identified chicken GnRH receptors (GnRHR1 and GnRHR2) are involved in mediating GnRH action on pituitary CART secretion, we examined their expression in the Ce and Ca lobes of chicken anterior pituitaries. *GnRHR2* mRNA could easily be detected in both lobes of anterior pituitaries by qPCR (Figure 1E), while only an extremely weak expression of *GnRHR1* mRNA was observed in the anterior pituitary (*data not shown*). This is similar to a previous report (Joseph et al., 2009); hence, it is likely that the action of GnRH on CART secretion is mediated by GnRHR2 predominantly expressed in chicken anterior pituitary.

### GnRH Induces CART mRNA Expression in Cultured Chick Pituitary Cells

**Figure 4:**
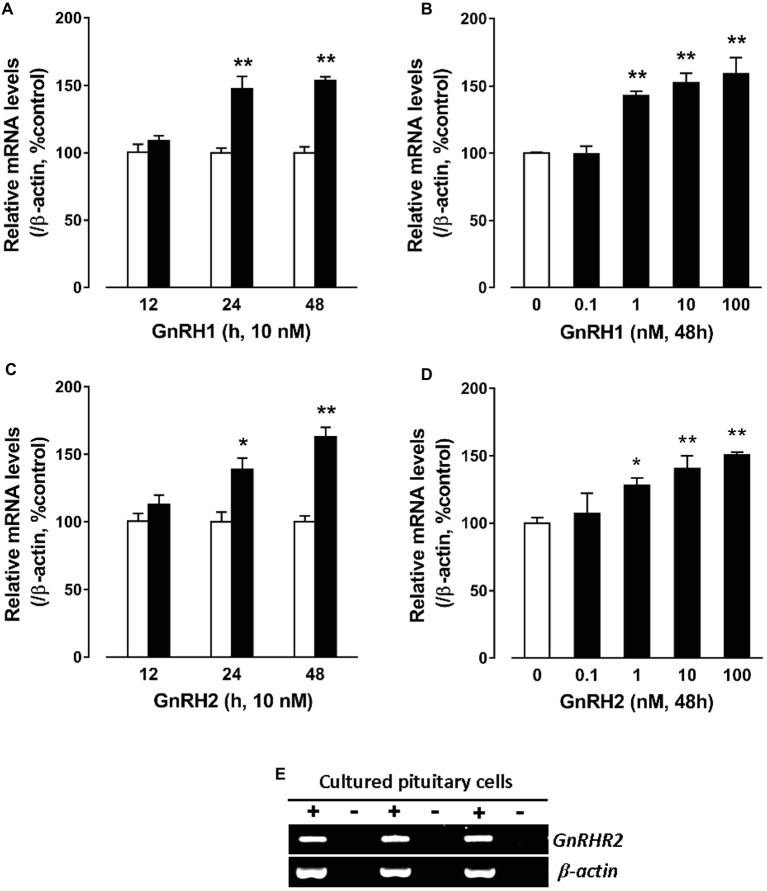
Quantitative real-time PCR (qPCR) assay showing the effects of gonadotropin-releasing hormone (GnRH) on cocaine- and amphetamine-regulated transcript (*CART*) expression in cultured chick pituitary cells. **(A,C)** Time- and **(B,D)** dose-dependent effects of chicken GnRH1 and GnRH2 on *CART* mRNA expression in cultured chick pituitary cells. Pituitary cells were incubated with GnRH1 (10 nM) or GnRH2 (10 nM) for the duration as indicated or with increasing doses (0–100 nM, 48 h) of GnRH1 or GnRH2 and subjected to qPCR assay of *CART* expression. The mRNA levels of *CART* were normalized by *β-actin* and expressed as the percentage of respective controls. Each value represents the mean ± SEM of four replicates (*N* = 4). ^*^p < 0.05, ^**^p < 0.01 vs. respective control. **(E)** RT-PCR detection of *GnRHR2* expression in cultured chick pituitary cells (three replicates). RT was performed in the presence (+) or absence (−) of reverse transcriptase. Parallel PCR of *β-actin* was performed to serve as an internal control.

To further examine whether GnRHs could regulate pituitary *CART* expression, the effect of GnRH1/2 on *CART* expression was examined in cultured chick pituitary cells by qPCR. As shown in Figure 4A, GnRH1 could induce *CART* expression in a time-dependent manner, and the maximal effect was observed after 48-h treatment. Moreover, GnRH1 could stimulate *CART* expression dose-dependently with the minimal effective dose noted at 1 nM (Figure 4B). Like GnRH1, GnRH2 could also induce *CART* expression in time- and dose-dependent manners (Figures 4C,D). In agreement with GnRH action on *CART* expression, GnRH receptor (*GnRHR2*, not *GnRHR1*) mRNA could easily be detected in cultured chick pituitary cells by RT-PCR (Figure 4E), supporting that GnRH-induced *CART* expression is likely mediated by GnRHR2.

### The Action of GnRH on Pituitary CART Expression and Secretion Are Mediated by Multiple Signaling Pathways

#### Signaling Property of Chicken GnRHR1 and GnRHR2

Although two GnRH receptors (GnRHR1 and GnRHR2) have been identified in chickens (Sun et al., 2001; Shimizu and Bedecarrats, 2006; Joseph et al., 2009), their downstream signaling pathways have not been fully characterized. Hence, in this study, we first examined the signaling property of chicken GnRHR1 and GnRHR2 using the three cell-based luciferase reporter assays established in our laboratory (pGL3-NFAT-RE-luciferase, pGL4-SRE-luciferase, and pGL3-CRE-luciferease reporter assay systems) (Wang et al., 2007, 2012; Mo et al., 2015), which are capable of monitoring receptor-stimulated calcium, MAPK/ERK, and cAMP/PKA signaling pathways, respectively.

**Figure 5:**
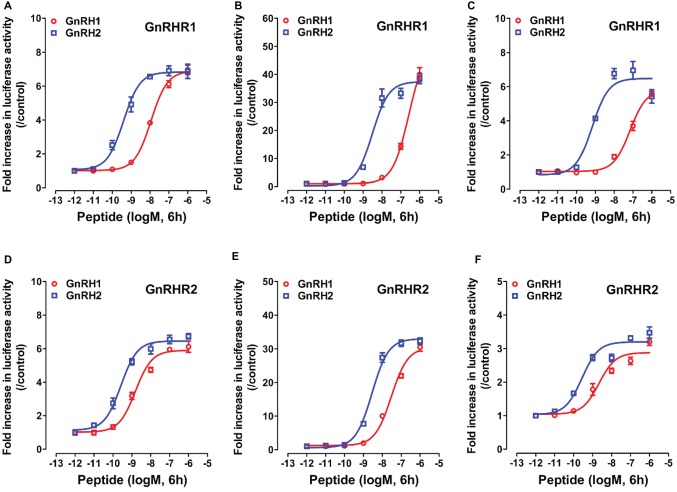
Activation of chicken GnRHR1 or GnRHR2 expressed in CHO cells by chicken GnRH1 or GnRH2, monitored by pGL3-NFAT-RE-luciferase **(A,D)**, pGL4-SRE-luciferase **(B,E)**, and pGL3-CRE-luciferease **(C,F)** reporter systems. CHO cells co-transfected with empty pcDNA3.1(+) vector and pGL3-NFAT-RE-luciferase (/pGL4-SRE-luciferase or pGL3-CRE-luciferease) reporter construct were used as internal controls, and peptide treatment did not alter the luciferase activity of CHO cells at any concentration tested (*data not shown*). Each data point represents mean ± SEM of four replicates (*N* = 4).

As shown in Figure 5, using the three cell-based luciferase reporter assays, we found that GnRHR1/GnRHR2 expressed in CHO cells could be activated by chicken GnRH1 and GnRH2, and thus stimulate the luciferase activities dose-dependently. This suggests that like mammalian GnRHR (Millar et al., 2004), both GnRHR1 and GnRHR2 are functionally coupled to the intracellular calcium, MAPK/ERK, and cAMP/PKA signaling pathways.

Interestingly, we found that GnRHR1 could be potently activated by GnRH2, but not by GnRH1 (Figures 5A–C), indicating that GnRHR1 is a receptor specific to GnRH2. Unlike GnRHR1, GnRHR2 could be potently activated by both GnRH1 and GnRH2 (with GnRH2 being slightly more potent than GnRH1) (Figures 5D–F), suggesting that GnRHR2 is a receptor common for both GnRH1 and GnRH2. The EC_50_ values of GnRH1 and GnRH2 in activating the two GnRHRs are listed in Table 2.

**Table 2:** EC_50_ values of chicken GnRH1 and GnRH2 in activating different signaling pathways of CHO cells expressing chicken GnRHR1/GnRHR2.

EC_50_ values (nM)		
Peptide	GnRHR1	GnRHR2
**Calcium signaling pathway**		
GnRH1	11.3	1.54
GnRH2	0.39	0.26
**MAPK/ERK signaling pathway**		
GnRH1	270	29.9
GnRH2	3.10	2.84
**cAMP/PKA signaling pathway**		
GnRH1	69.3	2.15
GnRH2	0.69	0.27

#### GnRH1-Induced CART Expression and Secretion Are Mediated by Multiple Signaling Pathways

Since GnRHR2 (and GnRHR1) are coupled to multiple signaling pathways (Figure 5), pharmacological drugs targeting the components of these signaling pathways were used to examine their involvement in mediating the actions of GnRH on pituitary CART expression and secretion.

**Figure 6:**
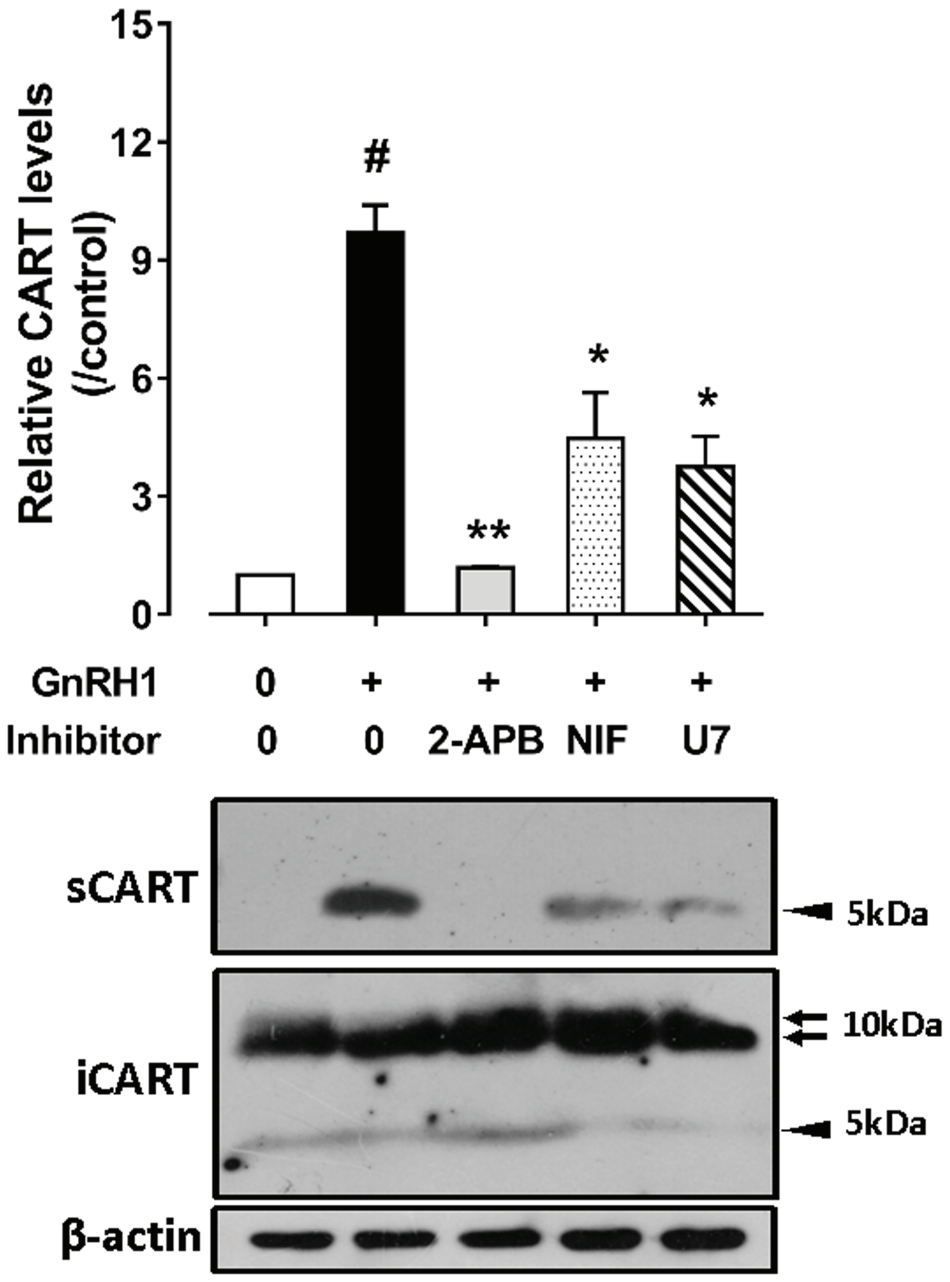
Western blot detected the effect of chicken GnRH1 (5 nM, 4 h) on cocaine- and amphetamine-regulated transcript (CART) secretion in the incubation medium of chick pituitaries incubated *in vitro* in the presence of 2-APB (100 μM), nifedipine (NIF, 5 μM), or U73122 (U7, 20 μM). The relative CART levels (sCART: ~5 kDa band) in the incubation medium were quantified by densitometry, normalized by that of β-actin band in pituitary tissue lysate, and then expressed as fold increase of controls without any treatment. iCART, intracellular CART, showed three major bands in pituitary tissue lysates. Each data point represents mean ± SEM of three replicates (*N* = 3). ^#^*p* < 0.001 vs. control; ^*^p < 0.05, ^**^p < 0.01 vs. GnRH1 treatment (in the absence of drug). One representative set of Western blotting is shown at the bottom.

**Figure 7:**
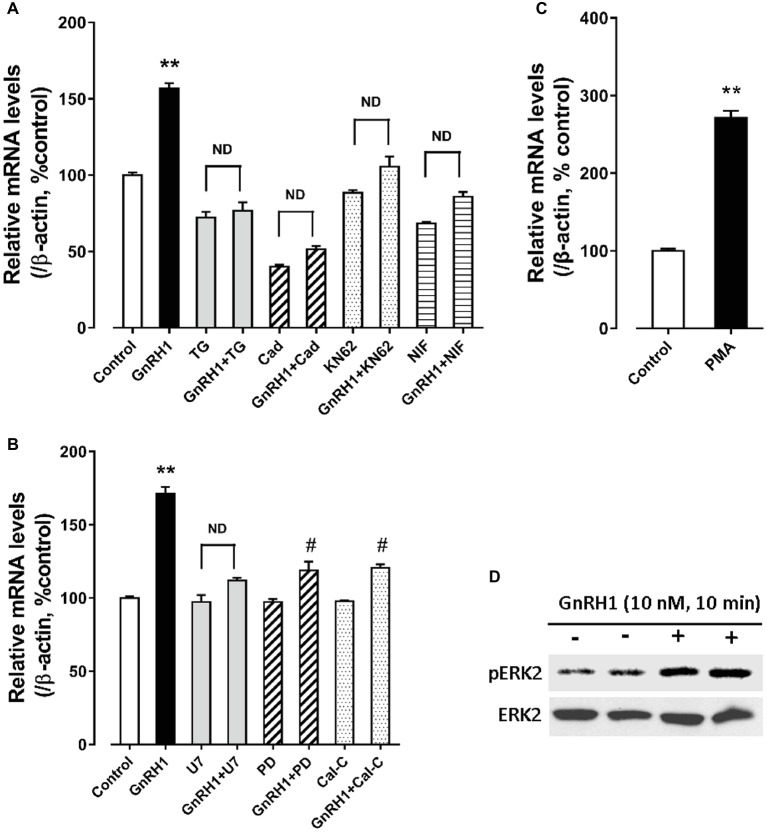
**(A)** Effect of GnRH1 (10 nM, 24 h) on cocaine- and amphetamine-regulated transcript (*CART*) mRNA expression in cultured chick pituitary cells in the presence or absence of thapsigargin (TG, 100 nM), calmidazolium (Cad, 1 μM), KN62 (5 μM) or nifedipine (NIF, 5 μM), respectively. **(B)** Effect of GnRH1 (10 nM, 24 h) on *CART* mRNA expression in cultured chick pituitary cells in the presence or absence of U73122 (U7, 20 μM), PD98059 (PD, 100 μM), or calphostin C (Cal-C, 100 nM), respectively. **(C)** Effect of PMA (1 μM, 48 h) on *CART* expression in cultured chick pituitary cells. The cells were treated for 24/48 h before RNA extraction and subjected to quantitative real-time PCR (qPCR) assay of *CART* mRNA levels. “ND” indicates no statistical difference between the two groups. Each data point represents mean ± SEM of four replicates (*N* = 4). ^**^p < 0.01 vs. control; ^#^*p* < 0.01 vs. GnRH1 treatment (in the absence of drug). **(D)** Western blot showed that GnRH1 treatment (10 nM, 10 min) could enhance ERK2 phosphorylation (pERK2) in cultured chick pituitary cells. Total ERK2 in cell lysates was also examined and used as internal controls. Note: only a single band for pERK2 or total ERK2 (42 kDa) was detected in cell lysate, because *ERK1* gene is lost in chicken genome (Gerilechaogetu et al., 2009). The representative sets of independent experimental duplicates are shown here.

As shown in Figure 6, GnRH1-induced CART secretion could be significantly inhibited by U73122 [a phospholipase C (PLC) inhibitor, 20 μM], 2-APB (a specific inhibitor of IP3 receptor, which can block IP3-induced calcium mobilization, 100 μM), and nifedipine (a calcium channel blocker, 5 μM), respectively, in intact pituitaries incubated *in vitro*, suggesting that GnRH1-induced CART secretion is likely mediated by PLC/IP3/Ca^2+^ signaling pathway and calcium channel coupled to GnRHR2. Moreover, we also found that U73122, thapsigargin [an inhibitor of sarco(endo)plasmic reticulum Ca^2+^-ATPase which can deplete intracellular calcium stores, 100 nM], calmidazolium (a calmodulin antagonist, 1 μM), KN62 [a specific inhibitor of Ca^2+^/calmodulin-dependent protein kinase II (CaMKII), 5 μM], and nifedipine (5 μM) could inhibit GnRH1-induced *CART* mRNA expression in cultured chick pituitary cells (Figures 7A,B). These findings suggest that PLC/IP3/Ca^2+^ signaling pathway and calcium channel coupled to GnRHR2 could also mediate GnRH1-induced *CART* mRNA expression.

Interestingly, we also found that calphostin C [a protein-kinase C (PKC) inhibitor, 200 nM] and PD98059 (an MEK inhibitor, 100 μM) could inhibit GnRH1-induced *CART* mRNA expression in cultured chick pituitary cells, while PMA, a PKC activator (100 nM), could mimic GnRH1 action (Figures 7B,C). These findings, together with the observation that GnRH1 treatment (10 nM, 10 min) could enhance ERK2 phosphorylation (42 kDa) in cultured chick pituitary cells (Figure 7D), suggest the involvement of PKC/MEK/ERK2 signaling cascade in mediating GnRH1-induced *CART* expression.

**Figure 8:**
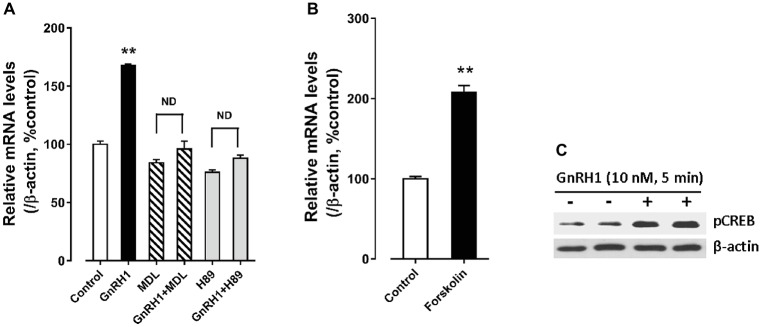
**(A)** Effects of MDL12330A (MDL, 20 μM) and H89 (10 μM) on GnRH1 (10 nM, 24 h)-induced *CART* mRNA expression. **(B)** Effect of forskolin (2 μM, 24 h) on basal *CART* mRNA expression. “ND” indicates no statistical difference between the two groups. Each data point represents mean ± SEM of four replicates (*N* = 4). ^**^p < 0.01 vs. control. **(C)** Western blot showed that GnRH1 (10 nM, 5 min) could enhance CREB phosphorylation (pCREB) in cultured chick pituitary cells. The β-actin in cell lysates were also examined and used as internal controls. The representative sets of independent experimental duplicates are shown here.

In addition, we found that forskolin [an adenlyate cyclase (AC) activator, 2 μM] treatment could mimic GnRH1 action on *CART* expression in cultured chick pituitary cells, while administration of either an AC inhibitor, MDL12330A (20 μM), or a specific PKA inhibitor, H89 (10 μM), could abolish GnRH1-induced *CART* expression (Figure 8). These findings, together with the demonstration that GnRH1 (10 nM, 5 min) could enhance CREB phosphorylation (43 kDa) in cultured chick pituitary cells (Figure 8), suggest that AC/cAMP/PKA/CREB signaling pathway is involved in GnRH1-induced *CART* expression.

## Discussion

In this study, we revealed that a large proportion of CART-ir cells is present in chicken anterior pituitaries, and GnRH stimulates CART secretion and expression *in vitro*. In addition, CART is present in the plasma at physiological level and GnRH can significantly elevate the circulating CART levels *in vivo*. To our knowledge, our study provides the first collective evidence that CART is a novel pituitary hormone, and its expression and secretion are controlled by the hypothalamic GnRH in chickens.

### Cocaine- and Amphetamine-Regulated Transcript (CART) Is a Pituitary Hormone in Chickens

In our previous study, we found that chicken CART peptide is predominantly and abundantly expressed in the anterior pituitary (Cai et al., 2015). In this study, using IHC and IF, we further demonstrated that CART-ir cells are densely distributed throughout the anterior pituitary and they made up ~12.3% of pituitary cells, which is comparable to the percentage of the other two hormone-producing cells (GH cells: ~12.8%; PRL cells: ~8.5%) (Figure 1; Mo et al., 2017). The extremely high expression level of CART in chicken anterior pituitaries is substantiated by our transcriptome analysis, in which *CART* is shown to be predominantly expressed in chick anterior pituitary, but not in the hypothalamus (Figure 1F). All these findings suggest that CART is a novel pituitary hormone in chickens. Similarly, *CART* mRNA and protein has been reported to be localized in the anterior pituitary in mammals (Thim et al., 1999; Murphy et al., 2000). In rodents, CART-ir signals are localized in several hormone-producing cells, including gonadotrophs, lactotrophs, and corticotrophs (Kuriyama et al., 2004; Stanley et al., 2004; Kappeler et al., 2006; Smith et al., 2006). Consistent with the findings in chickens and mammals, *CART* mRNA is reported to be expressed in goldfish pituitaries (Volkoff and Peter, 2001). Taken together, this suggests that CART may act as a pituitary hormone involved in the regulation of many physiological processes in birds and mammals, and possibly across other vertebrates as well.

In agreement with the idea that CART is a pituitary hormone in chickens, a remarkably high concentration of plasma CART peptide (1.2–3.5 ng/ml ≈ 0.2–0.7 nM) was detected in chickens at pre-pubertal and sexually mature stages, with the concentration peak noted at the latter sexually mature stage (Figure 1). Since CART is predominantly expressed in the anterior pituitary (Figure 1), the high circulating CART level is most likely contributed by this tissue. This is consistent with the findings in mammals, in which a high concentration of plasma CART peptide is present (~0.05–0.25 ng/ml in rats; 0.075–0.125 ng/ml in monkeys; 0.15–0.44 nM in humans) (Stanley et al., 2004; Vicentic et al., 2004; Kappeler et al., 2006; Bech et al., 2008) and contributed mainly by the anterior pituitary and secondarily by other peripheral tissues (e.g., ovary, gut, and fat) (Stanley et al., 2004).

The identification of CART peptide as a pituitary hormone in chickens, together with the evidence on gastrin-releasing peptide (GRP) being a potential pituitary hormone presented in our recent study (Mo et al., 2017), undoubtedly stresses the importance on revisiting the basic concept of avian pituitary biology, i.e., the avian anterior pituitary, which has long been thought to produce six classic hormones (GH, PRL, ACTH, TSH, FSH, and LH) (Proudman et al., 1999; Scanes, 2015), secretes additional endocrine hormones including CART and GRP peptides.

### GnRH1 Stimulates CART Secretion and Expression: Implications for the Roles of CART in Vertebrate Reproduction

In this study, we found that CART-ir cells have a spatial distribution similar to that of gonadotropins (e.g., LH), which are distributed throughout both cephalic and caudal lobes (Figure 1; Proudman et al., 1999; Scanes, 2015). This points to the possibility that like LH (Chou et al., 1985; Hattori et al., 1986; Sharp et al., 1986), CART secretion and expression may be controlled by hypothalamic GnRH. In agreement with this idea, we found that GnRH can potently stimulate CART secretion and expression *in vitro* (Figures 2, 4), and elevate the plasma CART levels *in vivo* (Figure 3). Our findings clearly indicate that besides hypothalamic CRH (Mo et al., 2015), GnRH is another potent stimulator of pituitary CART secretion and expression in chickens. Considering that the mRNA expression and pulsatile secretion of hypothalamic GnRH is reported to be negatively regulated by CRH in mammals (Kageyama, 2013), it is likely that in chickens, hypothalamic GnRH and CRH may regulate pituitary CART expression and secretion in a coordinated manner *in vivo*; however, this hypothesis still requires further elucidation.

**Figure 9:**
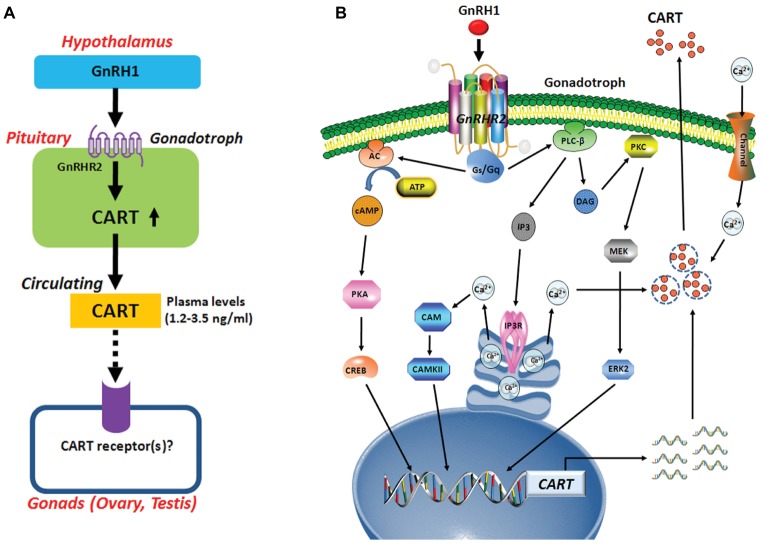
**(A)** Hypothetic model for gonadotropin-releasing hormone (GnRH)-induced pituitary cocaine- and amphetamine-regulated transcript (CART) expression and secretion, which may act on chicken gonads (ovaries/testes) *via* an endocrine route. In this model, CART is a novel pituitary hormone, and its secretion into the circulation is potently stimulated by hypothalamic GnRH1 (an ortholog of mammalian GnRH), suggesting that the development and functions of gonads (ovaries/testes) are likely controlled by CART peptide secreted from the anterior pituitary *via* CART receptor(s), which has not been identified in vertebrates. **(B)** The signaling pathways involved in mediating GnRH1-induced CART expression and secretion in pituitary gonadotrophs. In this model, GnRH1 stimulates CART expression and/or secretion *via* activation of GnRHR2 coupled to calcium channel and multiple signaling pathways including Gq-PLC/IP3/Ca^2+^, PKC/MEK/ERK, and Gs-AC/PKA/cAMP signaling pathways.

It is reported that CART peptide is co-localized with FSH and LH in rat gonadotrophs (Kuriyama et al., 2004; Kappeler et al., 2006). Although GnRH-induced pituitary *CART* expression has not been reported in any other vertebrate species before, a previous study suggested that CART secretion is induced from rat pituitary upon perfusion with GnRH (Kappeler et al., 2006). Hence, it is tempting to hypothesize that hypothalamic GnRH may play a conserved role in the control of pituitary CART expression and secretion, and the subsequent circulating CART level in birds and mammals (Figure 9A). Nevertheless, this theory has yet been substantiated in mammals.

There has so far been a research gap regarding the role of circulating CART peptide in birds and mammals. In this study, we noted that plasma CART levels are significantly higher in sexually mature chickens than in immature chicks (Figure 1D). This is in line with the highest mRNA level of *CART* detected in the anterior pituitary of sexually mature chickens in our recent study (Cai et al., 2015). Moreover, we demonstrated that GnRH can rapidly elevate plasma CART levels (Figure 3). Considering that GnRH is a pivotal player in the reproduction of birds and other vertebrate groups including the teleosts, frogs, reptiles, and mammals (Millar, 2005), it led us to speculate that circulating CART is likely involved in the control of reproduction, such as regulation of the development and/or functions of gonads (testis/ovary) *via* yet-to-be identified CART receptor(s) (Figure 9A; Sen et al., 2007; Rogge et al., 2008).

In mammals, there has been emerging evidence showing that CART may play a role in reproduction. In rodents, CART has been demonstrated to be a potent stimulator of both GnRH and kisspeptin (an up-regulator of GnRH neuron) neurons within the hypothalamus. CART-ir fibers are in close contact with GnRH cells and kisspeptin cells in rats, and this proximity may enhance both the amplitude and frequency of pulsatile GnRH secretion directly and indirectly (Lebrethon et al., 2000; de Roux et al., 2003; Irwig et al., 2004; True et al., 2013; Verma et al., 2014). In cows and ewes, CART is reported to be expressed in the ovary and present in ovarian follicular fluid (Kobayashi et al., 2004; Juengel et al., 2017). Moreover, *in vitro* experiments further supported that CART can inhibit basal and FSH-induced estradiol production in granulosa cells and may act as an important local autocrine/paracrine factor involved in the regulation of ovarian follicle development and steroidogenesis (Sen et al., 2007; Ma et al., 2016; Juengel et al., 2017). These pioneering findings, together with our convincing evidence showing that the expression and secretion of CART in chicken pituitary is regulated by GnRH (Figures 2–4), suggest that CART can act at different tier(s) of the hypothalamic-pituitary-gonadal axis (HPG) to regulate vertebrate reproduction (Kobayashi et al., 2004; True et al., 2013; Juengel et al., 2017).

### GnRH1-Induced CART Expression Is Mediated by Multiple Signaling Pathways Coupled to GnRHR2

In this study, we found that *GnRHR2* is abundantly expressed in chicken anterior pituitaries, whereas *GnRHR1* is almost undetectable. Our finding agrees with our transcriptome analysis showing the predominant expression of *GnRHR2* (not *GnRHR1*) in chick anterior pituitary (Figure 1F) and a recent observation that *GnRHR2* is abundantly expressed in chicken pituitaries (Joseph et al., 2009). All these findings indicate that GnRH1-induced CART secretion and expression is primarily mediated by GnRHR2 (and not GnRHR1) expressed in chicken anterior pituitaries (Figure 9A). Meanwhile, the potent effect of GnRH on CART secretion, together with the similar spatial distribution patterns of *CART*, *GnRHR2*, and *LHβ* within anterior pituitaries (Figure 1E), also suggests that CART peptide is likely localized in gonadotrophs (e.g., LH cells) expressing GnRH receptor (Figure 9), as in mammals (Kuriyama et al., 2004; Kappeler et al., 2006).

To elucidate the downstream signaling pathways coupled to GnRHR2 (and GnRHR1), using the three established cell-based luciferase reporter assays, we further examined the signaling properties of the two GnRH receptors. We demonstrated that like mammalian GnRHR (Millar et al., 2004), chicken GnRHR2 (and GnRHR1) expressed in CHO cells is functionally coupled to the calcium, MAPK/ERK, and cAMP/PKA signaling pathways (Figure 5). Our finding partially agrees with previous studies, in which activation of chicken GnRHR2 (and GnRHR1) expressed in COS-7 cells or GH3 cells can increase intracellular IP3 levels and may stimulate the cAMP/PKA signaling pathway (Shimizu and Bedecarrats, 2006, 2010; Joseph et al., 2009). Considering that chicken GnRH1 (an ortholog of mammalian GnRH), and not GnRH2, is present in the median eminence and directly involved in the control of LH secretion *via* GnRHR2 expressed in the chicken anterior pituitary (Sharp et al., 1990; Joseph et al., 2009), the high potency of GnRH1 in activating GnRHR2 (Table 2) also hints of the potential physiological importance of GnRH1-GnRHR2 signaling in the regulation of gonadotropin secretion and reproduction in chickens (Sharp et al., 1990; Joseph et al., 2009).

Using intact pituitaries or cultured pituitary cells, we demonstrated that Ca^2+^, PKC/MEK/ERK2, and AC/cAMP/PKA/CREB signaling pathways coupled to GnRHR2 could mediate GnRH1-induced CART expression and secretion, since inhibition or activation of these signaling pathways could block or mimic the GnRH1 action (Figure 9B). Our findings partially agree with the previous reports in mammals, in which the Ca^2+^ signaling pathway has been shown to regulate *CART* expression in pituitary cells or non-pituitary cells, *via* the use of pharmacological drugs including ionomycin and inhibitors of calmodulin and CaMKII (Jones et al., 2009). Moreover, PMA, a PKC activator, enhances *CART* mRNA expression in ovine pars tuberalis cells (Barrett et al., 2001). In addition, the *CART* mRNA levels are enhanced by an AC activator or a cAMP analog, and their stimulatory effect is abolished by H89 in rat GH3 cells (Barrett et al., 2001). All these findings support the involvement of intracellular PKC and AC/cAMP/PKA signaling pathways in the regulation of *CART* expression in birds and mammals.

In summary, we demonstrated that GnRH can potently stimulate pituitary CART secretion and expression, an action mediated by GnRHR2 coupled to Ca^2+^, PKC/MEK/ERK2, and cAMP/PKA signaling pathways (Figure 9). In line with this finding, GnRH1 can also elevate the plasma CART levels *in vivo*. To our knowledge, our study represents the first to build a clear concept that CART is a novel pituitary hormone and its expression, secretion, and plasma level are tightly controlled by hypothalamic GnRH in chickens. Evidence presented here also implies that CART peptide may play an active role in the HPG axis of vertebrate, which is a new facet of research that has long been neglected and is worthwhile exploring.

## Ethics Statement

This study was carried out in accordance with the recommendations of the guidelines provided by the Animal Ethics Committee of Sichuan University. The protocol was approved by the Animal Ethics Committee of Sichuan University.

## Author Contributions

CM and YW conceived and designed the experiments and analyzed the data. CM, CL, LH, ZL, and JZ performed the experiments. CM, JL, and YW contributed reagents, materials, and analysis tools and wrote the paper.

### Conflict of Interest Statement

The authors declare that the research was conducted in the absence of any commercial or financial relationships that could be construed as a potential conflict of interest.

## Footnotes

^1^http://www.ensembl.org/Gallus_gallus
